# Visualization and Improvement of the Quality, Efficiency, and Equity of the Healthcare System - Secondary Publication

**DOI:** 10.31662/jmaj.2023-0196

**Published:** 2024-04-11

**Authors:** Yuichi Imanaka

**Affiliations:** 1 Department of Healthcare Economics and Quality Management, School of Public Health, Graduate School of Medicine, Kyoto University, Kyoto, Japan

**Keywords:** Health Care System, Quality of Care, Efficiency of Care, Equity of Care, Social System for Health, Big Data Analysis, Health Policy and Management, Health Care Reform

## Abstract

In a depopulating society, it is difficult to ensure sufficient resources and finances for health and health care. Thus, effective management of the reform of the healthcare system by visualizing the quality, efficiency, and equity of health care is imperative. This article presents an overview of the studies conducted by my team in this area over the past 35 years, covering the following four sections: (1) visualization of healthcare system using individual-level data, (2) healthcare system at the organizational level, (3) healthcare system at the national and regional levels, and (4) creation of a social system for health.

To improve the quality, efficiency, and equity of the healthcare system as well as the social system for people’s health, it is necessary to visualize the actual situation and share this information with all stakeholders to contribute to the joint management of healthcare system. On this basis, from the perspectives of each region and the nation, it is important to visualize and grasp various wider determinants of people’s health and healthcare performance and to improve health care and social systems.

The aim of the healthcare system is to achieve higher quality, efficiency, and equity in health care and to secure and improve people’s health. A depopulating society faces difficulties in ensuring resources and finances; thus, it is imperative to effectively manage the reform of the healthcare system. To this end, it is essential to visualize the quality, efficiency, and equity of health care. An overview of the developments in this area over the past 35 years is presented as follows, with some example papers shown by PMID in the parenthesis, in addition to the cited literature.

## 1. Visualizing Healthcare System Using Individual-Level Data

To visualize the quality, efficiency, and equity of health care, we must first overcome the challenges of quantifying the quality, including access to appropriate care. Only after quality is quantified can efficiency be captured in contrast to costs. Then, equity can be eventually captured by showing the disparities in quality and efficiency.

In 1995, I led the launch of the project Quality Indicator/Improvement Project (QIP) with 10 volunteer hospitals, aiming to measure and improve the quality and efficiency of health care in total and in each area of patient classifications through joint efforts to standardize the routinely collected dataset. The dataset included patients’ basic characteristics, diagnoses, and procedures with common coding systems, disease severity information, as well as dates and routes of admission and discharge. This framework was derived from the harsh experience of putting enormous efforts in extracting necessary data elements from medical records, which were paper-based and nonstandardized at that time. Over 500 hospitals all over Japan have registered to participate in the QIP. Based on the QIP, I showed the importance of a patient casemix classification system and developed its prototype for measuring the quality and efficiency of health care and for improving management and policy ^(1)^.

The QIP contributed to the Japanese DRG/PPS Pilot Project of the Ministry of Health, Labour and Welfare (MHLW), which served as the predecessor of DPC (“Diagnosis-Procedure Combination”: one of the patient casemix classification systems) (1998-present). In addition, it contributed to the launch of the MHLW’s DPC in 2001 by offering the patient classification prototype, analysis experience, and dataset forms, including EF-file formats. I had committed myself here since the preparation stage, became a core team member on DPC Research & Development, and then opened up the path for clinical research and health services research using DPC data (published in 2002, PMID: 12389807, 15212865, 15375097, 15212865, 15770381, etc.).

**Figure 1.**
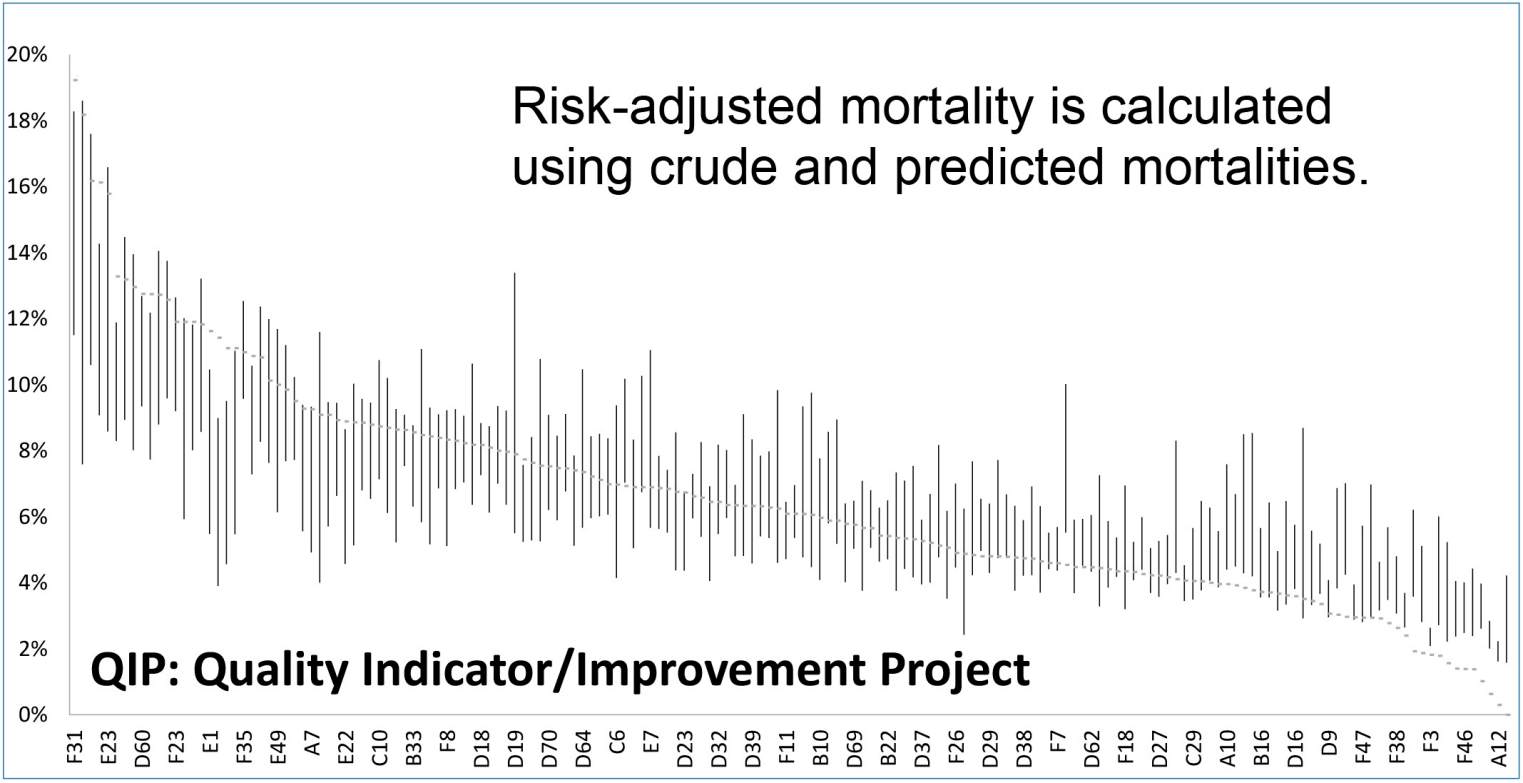
Crude and Predicted Mortalities of Patients with Acute Myocardial Infarction at the Hospital Level.

For outcome evaluation, we have developed a highly accurate model to predict outcomes, e.g., mortality (Figure 1), produced Risk-Adjusted Outcomes, and refined how to use comorbidity information ^(2)^ to improve risk adjustment. For process evaluation, we have developed a large number of process quality indicators in various clinical areas in the QIP. Most of these indicators measure process concordance with evidence-based recommendation. Then, we have been working to understand the causes of quality variations among hospitals ^(3)^. This project was the first to establish a multicenter benchmarking system in Japan, which became the cornerstone of various subsequent healthcare quality indicator projects.

Furthermore, we promoted the use of administrative databases of healthcare claims, long-term care (LTC) claims, and health checkups (PMID: 33544396, 31623988, 29626553, etc.), so-called Big Data, and advanced into the integrated use by linking the above three databases (e.g., prediction of the increasing level of LTC need ^(4)^, impact of LTC copayment increase on health care and LTC costs ^(5)^, and prediction of worsening diabetes ^(6)^).

For costing, I led the Health Care Costing project funded by the MHLW to develop a standardized method for costing ^(7)^, and I actually measured costs and their compositions based on DPC/patient classifications and clinical areas/clinical disciplines, cost variations, and correspondence between medical fees and costs (PMID:19522724, etc.). The developed costing method also became an important foundation for cost accounting systems of various hospital-wide integrative medical information systems (PMID: 19522724, etc.).

Furthermore, amid a rapidly increasing and exhausting workload for patient safety, particularly between 2000 and 2010, we quantified the increased burden on healthcare institutions caused by patient safety i.e., the Cost of Safety (Cost of Quality) (PMID: 18514966, etc.). It led to the conduct of national-level survey by the Central Social Insurance Medical Council (*Chu-i-kyo*), where I was in charge of the design and analysis. The results were reported sequentially from 2005 to 2008 and served as input for the additional medical fee points of patient safety measures.

## 2. Healthcare System at the Organizational Level

The successful care delivery of a healthcare organization is at the core of the performance of the overall healthcare system. It is important to visualize the healthcare performance at the organizational level, including the aforementioned hospital Quality Indicators, and to build a foundation for quality improvement.

Since the early 1990s, we have pioneered research on patient satisfaction and experience, i.e., Patients’ Assessment of Quality of Care, and Organizational Culture of healthcare providers by developing highly reliable and valid evaluation methods through continuous and repetitive multicenter surveys. The study results were highly praised at international conferences as a pioneering demonstration of the association of organizational culture with patient satisfaction and, in turn, with healthcare quality indicators ^(8)^. These surveys have been used to improve the healthcare delivery system at many hospitals and are currently undergoing further research and development.

As an executive director in charge of research and development at the Japan Council for Quality in Health Care, I was responsible for the development and implementation of new evaluation standards and methods for Patient Safety Management and Clinical Care Process in the Hospital Accreditation System since 2000. Subsequently, these two areas (Patient Safety Management System and Clinical Care Process) have become increasingly important and now comprise the main part of the accreditation scheme.

With regard to human resource development, I was in charge of the R&D project to establish the Board Certification System of Physicians for Public Health and Social Medicine, which was funded by the MHLW under the Comprehensive Community Health Promotion Project for fiscal years 2015-2016. This focused on the concept development, concept realization, joint operation of stakeholders, and formation of policies, criteria, procedures, and management systems for board certification ^(9)^. This specialist certifying system was successfully started in April 2017 by the Japan Board of Public Health and Social Medicine, a new agency managed jointly by eight academic societies and six associations, including the Japan Medical Association and the Japanese Medical Science Federation. Here, Health Crisis Management is newly introduced as one of the core areas in the specialist training system. The specialist training program committee is comprised of supervisors from the four areas of local or national governments, occupational health institutes, healthcare organizations, and universities, strengthening collaboration among stakeholders in training and practice. This four-area collaboration actually worked well in COVID-19 countermeasures.

## 3. Healthcare System at the National and Regional Levels

Healthcare systems and policies are basically designed and implemented at the national level, but visualization is also important in understanding the overall picture, setting strategic goals, and planning. In future healthcare systems, the key factors are visualization, information sharing, and collaboration in “local” settings, aiming at overall optimization and resilience enhancement.

As the Principal Investigator for the Western Pacific Region of the joint project of the International Hospital Federation and World Health Organization, I led and conducted a comparative study of Health Care and Hospital Reforms in various countries Worldwide ^(10)^. More recently, we conducted a comparative analysis of Japanese and UK policies on Antimicrobial Resistance (AMR) control, which has been a global political issue (PMID: 30369423), made policy recommendations for AMR research promotion with European researchers (PMID: 34557847), and have been involved in AMR policy research in Japan (PMID: 33639873, 28654675, etc.). During the COVID-19 Pandemic in 2020, we demonstrated the need for public assistance for healthcare organizations from the side of academia first, and then we quickly assessed and published the impacts of COVID-19 on various aspects of the healthcare system, including a dozen of papers in 2020-2021 on hospital management and clinical care as well as on pneumonia ^(11)^, bronchial asthma ^(12)^, surgery in various areas ^(13)^, and alcohol-related diseases ^(14)^.

**Figure 2.**
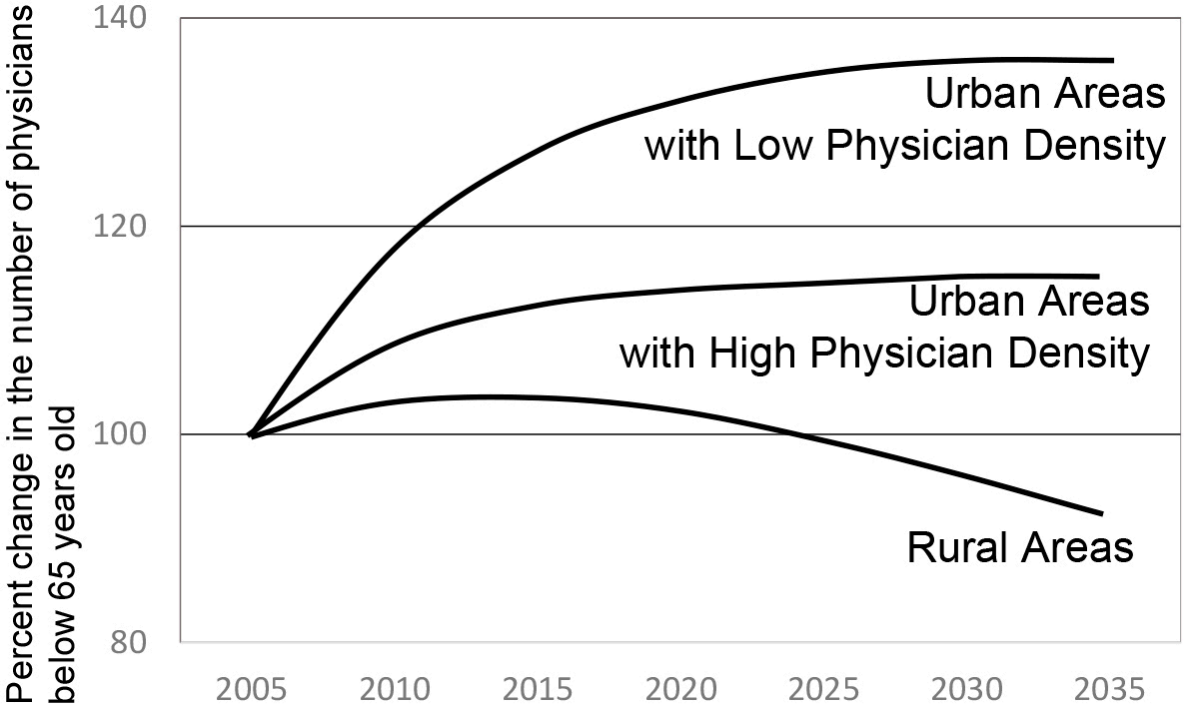
Future projection of the number of physicians in urban and rural areas Note) The details should be referred to the previous study ^(15)^ on which the above figure is created.

On the health care Resource Allocation, we quantitatively showed that the disparity will further widen in the future if the current situation continues (Figure 2) ^(15)^, sounding the alarm over the worsening maldistribution of physicians (PMID: 30224401). For physician supply in each medical specialty, we visualized large demand and supply gaps in health care . Furthermore, we advocated the need to adjust the number-of-physician indicator based on the demand (PMID: 29317415). Subsequently, the MHLW began to adjust to the demand and presented a new indicator of physician maldistribution.

**Figure 3.**
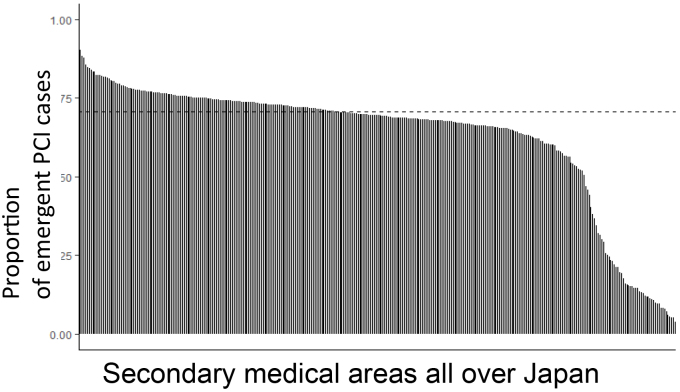
Proportion of emergent PCI cases of acute myocardial infarction at the secondary medical area all over Japan（Regional QI： FY2020） ^*^Calculated using the National Database (NDB) of claims data of the Ministry of Health, Labour and Welfare. 2022.

Regarding regional healthcare systems, a project was started at the Kyoto Medical 'Anshin' Health Care System Project in 2009 with studies visualizing and analyzing healthcare performance and resources at the regional level, and it developed and measured healthcare quality indicators for each region, such as the secondary medical care area (PMID: 25444024). As of 2022, by using the nationwide healthcare claims data NDB, we have developed Quality Indicators at the Regional Level (Figure 3) and studied its causal structures to contribute to discussion in policy plans such as regional healthcare plans (e.g., doi: https://doi.org/10.1101/2022.05.20.22275402).

Securing resources and finance for healthcare system in a super-aging depopulating society is expected to become increasingly difficult. Here, we need “Social Joint Venture” ^(16)^ to overcome these difficulties. Based on the major activities of healthcare professionals, all stakeholders, including governments, citizens, insurers, mass media, academia, education, various industries, and social entrepreneurs, should share information on actual situations and consciously and explicitly cooperate to proactively play their unique roles for the collaborative management of the healthcare system. This is what I propose as “Social Joint Venture”^(16)^. Here, civil empowerment is the key for success. With limited resources and finance, a high-quality, efficient, and equitable healthcare system cannot be sustained by leaving it to market principles. Instead, it should be proactively built through all the stakeholders’ joint work mobilizing human wisdom and resources.

## 4. For the Creation of a Social System for Health

The ultimate goal of the healthcare system is to assure and improve the health of the people. For this purpose, in addition to the healthcare system, socioeconomic environment, behavior and lifestyle, and natural and built environment (so-called wider determinants of health) are becoming increasingly important as targets to improve health. When I was a medical student, I developed and presented a new model of health determinants, i.e ., “Health Support Model” at the Annual Conference of the Japanese Society of Public Health (1985 the Society Journal - Supplement I [abstract book], p.6), which already incorporated the so-called wider determinants of health, including human and socioeconomic factors/environments (e.g., social participation and interaction, social support, natural and built environment) and individual factors (e.g., biological assets, skills, self-efficacy, knowledge, attitudes, behaviors).

To comprehensively approach wider health determinants, we formed a multidisciplinary unit formalized in our university called Value Creating Design Hub for Super-Ageing Societies (preparatory activities since 2014, inaugurated in 2016, awarded with Age-Tech Award in 2021). Based on this multidisciplinary unit, we formed an Industry-Academia-Government Consortium, PEGASAS [open-innovation Platform of all-area Enterprises, Governments, and Academia to design and realize Super-Aging Societies] (2017-present) to promote research on various social designs and urban/rural development. Through this industry-academia-government consortium, a Structural Model of Healthy Smart City ^(17)^ was developed to set the vision and comprehensively assess the status of wider determinants of health of each region.

Simultaneously, we have conducted development and research of various indicators of healthy life expectancy and actually measured such indicators in municipalities nationwide using Big Data. It is also planned to provide feedback to all regions of the country regarding the information on the health conditions and the wider determinants of health to comprehensively grasp each region and to Improve Societal Environment For Health.

These informatization efforts are associated with the Council on Competitiveness-Nippon, under Keidanren (COCN ) Technology Policy Proposals in 2017 and 2018, which I led, to construct healthy smart cities through digital transformation and industrial creation, consequently associated with the COCN smart city proposal in 2019.

## 5. Conclusion

To improve the quality, efficiency, and equity of the healthcare system as well as those of the social system for people’s health, it is essential to visualize the actual situation and share this information with all stakeholders to contribute to the joint management of the healthcare system. On this basis, from the perspectives of each region and the nation, it is imperative to visualize and grasp the various wider determinants of people’s health and healthcare performance and to improve healthcare and social systems.

## Article Information

This article is based on the study, which received the Medical Award of The Japan Medical Association in 2022. This is a revised English version of the article originally published in Japanese in the Journal of the Japan Medical Association 2023;151(10): 1824-8 ^(18)^. The original version is available at https://med.or.jp/cme/jjma/newmag/pdf/151101824.pdf. Only members of the Japan Medical Association are able to access it.

### Conflicts of Interest

None

### Acknowledgement

I would like to express my deepest gratitude to all those who have supported and contributed to the above research, development, and implementation efforts.
